# High frequency of Machado-Joseph disease identified in Southeastern Chinese kindreds with spinocerebellar ataxia

**DOI:** 10.1186/1471-2350-11-47

**Published:** 2010-03-25

**Authors:** Shi-Rui Gan, Sheng-Sheng Shi, Jian-Jun Wu, Ning Wang, Gui-Xian Zhao, Sheng-Tong Weng, Shen-Xing Murong, Chuan-Zhen Lu, Zhi-Ying Wu

**Affiliations:** 1 Department of Neurology and Institute of Neurology, Huashan Hospital, State Key Laboratory of Medical Neurobiology, Shanghai Medical College, Fudan University, 12 Wulumuqi Zhong Road, Shanghai 200040, China; 2 Department of Neurology and Institute of Neurology, First Affiliated Hospital, Center of Neuroscience, Fujian Medical University, 20 Chazhong Road, Fuzhou 350005, China

## Abstract

**Background:**

Machado-Joseph disease (MJD), caused by a CAG repeat expansion located in exon10 of the *ATXN3 *gene, is now regarded as one of the most common spinocerebellar ataxia (SCA) in the world. The relative frequency of MJD among SCA has previously been estimated at about 50% in the Chinese population and has been reported to be related to the frequency of large normal alleles in some populations. Taq polymerase has been used for PCR in nearly all studies reported previously.

**Methods:**

Normal and expanded alleles of *ATXN3 *were detected via PCR using LA Taq DNA polymerase (better for GC-rich sequences) and denaturing polyacrylamide gel electrophoresis in 150 normal individuals and 138 unrelated probands from autosomal dominant SCA families. To compare reaction efficiency, 12 MJD patients' expanded alleles were amplified with La Taq and Taq polymerase respectively in the same amplifying systems and reaction conditions.

**Results:**

Normal alleles ranged from 12 to 42 CAG repeats. The most common allele contained 14 repeats with a frequency of 23.3%, which corroborates previous reports. The frequency of large normal alleles (>27 repeats) was 0.28, which was very high relative to previous reports. The frequency of MJD in SCA patients was 72.5%, which was significantly higher than those in previous reports about the Chinese and other Asian populations. This frequency was one of the highest reported worldwide, with only Portuguese and Brazilian populations exhibiting higher proportions. All 12 expanded alleles were amplified in PCR with La Taq polymerase, whereas only 2 expanded alleles were amplified with Taq polymerase.

**Conclusion:**

We have first reported the highest relative frequency of MJD in Asia, and we attribute this high frequency to a more efficient PCR using LA Taq polymerase and hypothesized that large ANs may act as a reservoir for expanded alleles in the Southeastern Chinese population.

## Background

Machado-Joseph disease (MJD), also called spinocerebellar ataxia type 3 (SCA3), is associated with a variety of clinical manifestations, including progressive ataxia, ophthalmoplegia, a variable degree of pyramidal signs, extrapyramidal signs and facial myokymia [1]. MJD was initially reported in Portuguese-Azorean descendants and is now regarded as one of the most common spinocerebellar ataxia (SCA) in the world [2,3]. It is caused by a CAG repeat expansion located in exon10 of the *ATXN3 *gene on chromosome 14q32.1 [4]. The number of CAG repeats was first described as 13-36 in normal alleles (ANs) and 68-79 in expanded alleles. With continued research and publication of related data, the range of ANs has broadened to 12-44, and now MJD is usually molecularly diagnosed when the CAG repeat crosses a threshold of 52 without regard for whether some patients carry "intermediate alleles" or "reduced penetrance alleles", such as 45-51[5].

The most common method used to study the expanded alleles of *ATXN3 *is to amplify expanded alleles with MJD52/MJD25 or MJD52/MJD70 primers [4]. Although amplifying systems and ion conditions vary across studies, Taq polymerase was used for PCR in nearly all previous reports. The efficiency of amplification is affected by long length and high GC content in the amplified sequence when using Taq polymerase [6] and it is difficult to amplify expanded alleles having large CAG repeats [7]. To alleviate this, here we use the LA Taq polymerase which is better for amplifying expanded alleles with GC-rich sequences.

The frequency of MJD in SCA patients differs in different population, and some studies [8-15] reported that the relative frequency of dominant SCA (including MJD) was related to the frequency of large normal alleles in some populations. In the present study, we have recruited 150 unrelated healthy individuals and 138 probands from autosomal dominant SCA families of Southeastern Chinese origin to analyze the distributions and characteristics of CAG repeats of *ATXN3*, and have found a much higher frequency (72.5%) of MJD in SCA patients compared to previous reports. In fact, this relative frequency is only slightly less than that reported in the Portuguese and Brazilian populations (84.2%) [16,17]. We have attributed this high relative frequency to better detection of expanded alleles via more efficient PCR using LA Taq polymerase and hypothesized that large ANs may act as a reservoir for expanded alleles in the Southeastern Chinese population.

## Methods

### Subjects

**Figure 1:**
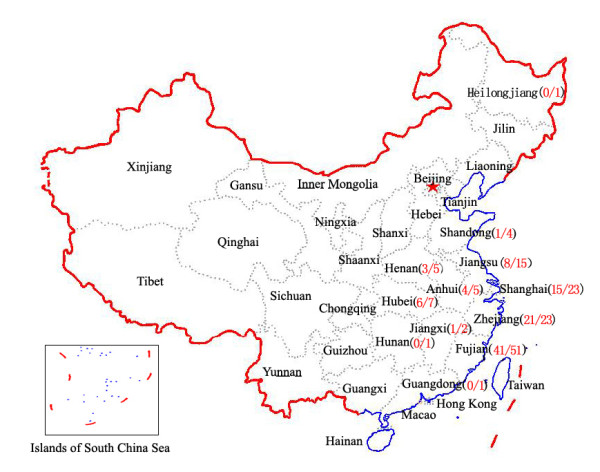
**Distribution of the SCA families in the present study**. The numbers in parentheses indicate numbers of MJD families/numbers of SCA families. (Reproduced with permission from http://nfgis.nsdi.gov.cn)

One hundred and fifty unrelated healthy individuals and 138 unrelated probands from autosomal dominant SCA families were recruited from the Southeastern Chinese population (figure 1) between July 9, 2003 and June 31, 2009. Patients were clinically diagnosed in accordance with the previously published standard [18]. Each patient was given detailed clinical and neurological examinations by two experienced neurologists and a clinical history was obtained. Informed consent was obtained from each subject (if <18 years of age, consent was obtained from their legal guardians) and the protocol was approved by the ethical committee. Genomic DNA was extracted from peripheral EDTA blood via the salt precipitation method [19] or with a QIAamp DNA Blood Minikit (QIAGEN, Hilden, Germany).

### Molecular analysis

The CAG repeat expansion located in exon 10 of *ATXN3 *was amplified using MJD52/MJD25 primers as described in the previous report [4]. The PCR amplification was performed in a total volume of 25 μL containing 0.10 μg of genomic DNA, 0.10 μmol/L of each primer, 50 μmol/L of each dNTP and 1.25 units of LA Taq polymerase with 12.5 μL 2× GC buffer I (TaKaRa, Chiba, Japan). PCR products were generated using a Mini-Cycler PCR system (Applied Biosystems, Foster City, CA, USA). After an initial denaturation for 2 minutes at 94°C, the PCR reaction included 30 cycles of denaturation at 94°C for 30 seconds, annealing at 58°C for 45 seconds and extension at 72°C for 1 minute, followed by a final extension at 72°C for 5 minutes. PCR products were separated by an 8% polyacrylamide gel that was run at 27 V/cm at 55°C for 3 hours. The pGEM-3Zf (+) DNA-Hae III marker was used as a DNA size marker and the gel was silver stained to visualize the bands. Based on the marker size (bp) and the transport ratio of the DNA marker in the gel, we used a statistical package (version 11.0, SPSS, Chicago, IL) to generate a curve with the equation Y = aX^b ^(*Y *indicated the size of fragments of PCR products, *X *indicated the transport ratio of fragments of PCR products, *a *and *b *indicated coefficient and exponent that all generated by the statistical package, respectively) to estimate size of PCR products. The estimated number of CAG repeats = (size of fragments of PCR products- 161bp)/3. The numbers of CAG repeats of all subjects were estimated in this way at first. To verify accuracy of CAG numbers estimated, 10 normal alleles which were estimated to contain 14 CAG repeats and all expanded alleles were further confirmed by sequencing. The PCR products were electrophoresed on a 2.5% agarose gel and separated bands were excised. The DNA contained in excised bands was purified from the gel using the Geneclean II Kit (Qbiogene, Carlsbad, CA, USA). The purified product was sequenced using the procedure which has been described elsewhere [20].

To compare the efficiency of amplification using La Taq and Taq polymerase, 12 MJD patients' expanded alleles which were confirmed by PCR using La Taq polymerase were amplified with Taq polymerase in the same amplifying systems and reaction conditions mentioned above except that LA Taq polymerase with 12.5 μL 2× GC buffer I was displaced by Taq polymerase with 2.5 μL 10× buffer (Dichuan Inc, Shanghai, China). The PCR products amplified with Taq and La Taq polymerase were all electrophoresed on 2.5% agarose gel. The DL2000 (Tiangen, Bejing, China) was used as a DNA size marker.

### Statistical analysis

All statistical analyses were performed using SPSS software version 11.0 (SPSS, Chicago, IL). The mean, median, variance and skewness were determined for the distributions of ANs. In accordance with the previous report[9], the alleles carrying more than 27 CAG repeats (>27 repeats) were defined as large ANs. Chi-square tests were used to analyse the difference between present study and other studies both in the frequency of the large ANs and the relative prevalence of MJD. The results were considered statistically significant at *p *< 0.05.

## Results

### Analysis of CAG repeats in normal individuals

**Figure 2:**
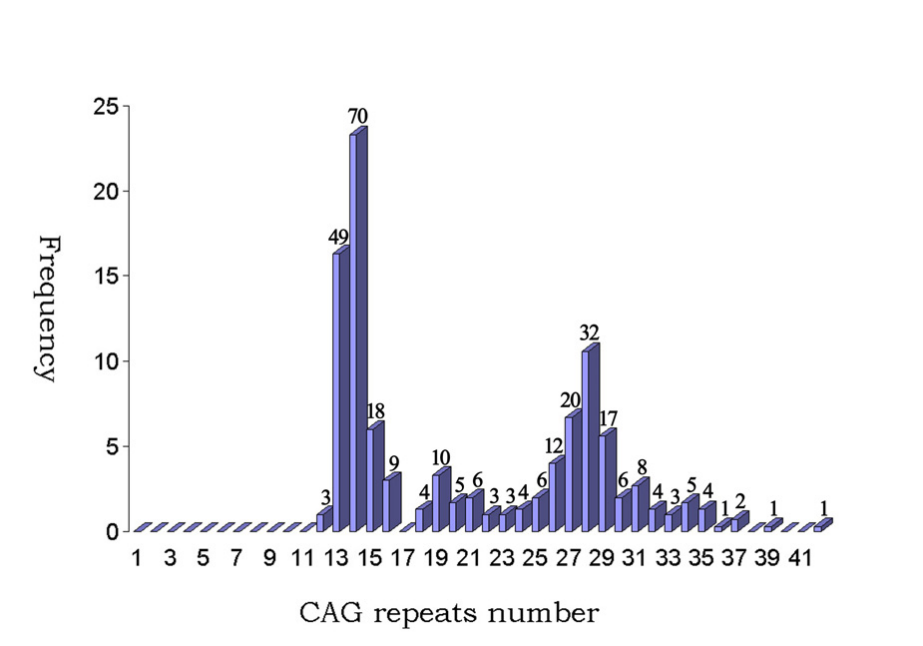
**Distribution of the CAG repeats in 300 ANs from 150 healthy Chinese individuals**. The numbers upon the column indicate the number of allele.

**Figure 3:**
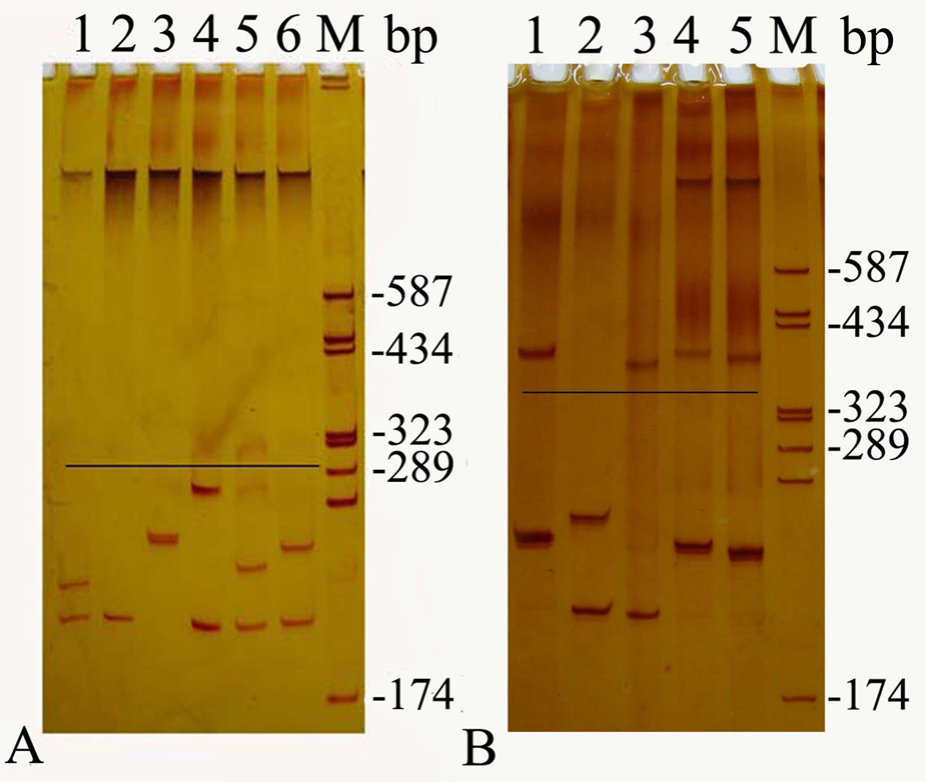
**The polyacrylamide gel electrophoresis analysis of ANs (A) and alleles of SCA patients (B)**. In figure 2A, lanes 1-5 were normal individuals. The black line indicates the upper limit of ANs. In figure 2B, lanes 1, 3, 4, and 5 were MJD patients; lane 2 was an SCA patient, but not a MJD patient. The black line indicates the place of the lower limit of expanded alleles. M: pGEM-3Zf (+) DNA-Hae III marker.

**Figure 4:**
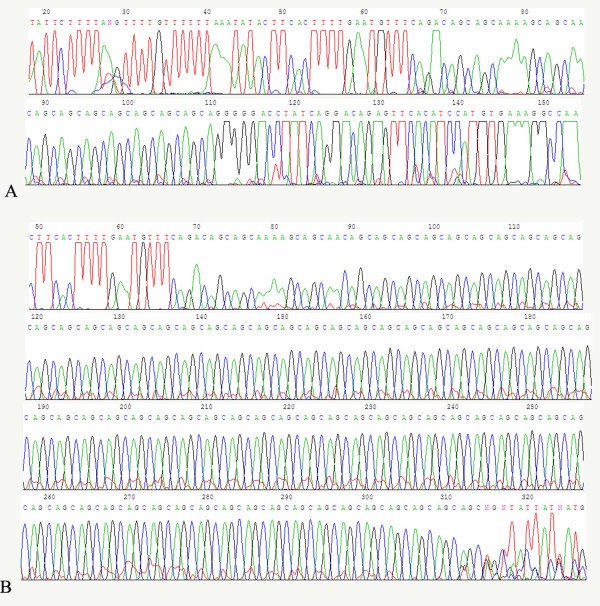
**Chromatograms of the AN with 14 CAG (A) and the expanded allele with 81 CAG (B)**.

Twenty-seven alleles with the heterozygosity of 0.78 were identified in 150 normal individuals. The distribution of the 27 alleles is shown in figure 2. The number of CAG repeats ranged from 12 to 42. The three most frequent alleles had 14 (23.3%), 13 (16.3%) and 28 (10.6%) CAG repeats. The mean, median, variance and skewness were 20.8, 19.0, 56.0 and 0.42, respectively. The polyacrylamide gel electrophoresis analysis is shown in figure 3A and the results for the individual who was carrying homozygous for an allele which was estimated to contain 14 CAG repeats was confirmed by sequencing (figure. 4A). The difference in the frequency of large ANs between the present study and other studies involving Japanese [9], Indian [10], Czech [11] populations and a combined population comprised of Acadian, Black, Caucasian, Inuit and Thai [21] is shown in table 1.

**Table 1:** The frequencies of large normal alleles of *ATXN3 *gene

Number of CAG repeats	Present study	Japanese			Indian			Czech			Combined Population		
	
	Frequency	Frequency	χ^2^	P	Frequency	χ^2^	P	Frequency	χ^2^	P	Frequency	χ^2^	P
>27	0.28	0.21	3.683	0.055	0.12	42.045	0.000	0.09	27.663	0.000	0.17	8.616	0.003
>28	0.19	0.11	6.788	0.009	0.08	29.060	0.000	0.04	21.956	0.000	0.11	10.341	0.001
>29	0.13	0.07	5.869	0.015	0.04	28.517	0.000	0.04	11.836	0.001	0.08	5.264	0.002
>30	0.10	0.05	4.342	0.037	0.03	22.329	0.000	0.03	8.499	0.004	0.07	3.192	0.074
>31	0.07	0.05	1.332	0.248	0.02	14.120	0.000	0.01	8.186	0.004	0.05	2.190	0.139

### Analysis of MJD expanded alleles

One hundred out of 138 probands were identified as having one expanded allele (>60 repeats), with the numbers of repeats varying from 68 to 84. Thus, the frequency of MJD in 138 SCA families was 72.5% (100/138), which is significantly higher than those reported in other Asian populations (table 2) and is quite high worldwide, being lower only than the Portuguese and Brazilian populations (table 3). In the other 38 probands with no expanded allele, 6 of them were homozygous for normal alleles and need to be further studied by other techniques. The polyacrylamide gel electrophoresis analysis is shown in figure 3B and the chromatogram of an expanded allele with 81 CAG repeats is shown in figure 4B.

**Table 2:** Frequencies of MJD in SCA patients from different Asia populations

	Number of MJD	Number of SCA	Frequency (%)	P	Reference
**Present study**	**100**	**138**	**72.5**		

Chinese mainland	59	120	49.2	<0.001	23
	41	85	48.2	<0.001	24
	26	75	34.7	<0.001	25
	1	8	12.5	<0.001	22
Chinese Taiwan	35	74	47.3	<0.001	26
	26	81	32.1	<0.001	27
Singapore	15	36	41.7	0.001	28
Japan	87	202	43.1	<0.001	9
	12	29	41.4	0.001	29
	91	330	27.6	<0.001	30
	30	113	26.5	<0.001	31
	35	143	24.5	<0.001	32
	28	117	23.9	<0.001	33
	8	46	17.4	<0.001	34
	3	86	3.5	<0.001	35
Thailand	35	182^#^	19.2	<0.001	36
Korea	4	40	10.0	<0.001	37
	13	237	5.5	<0.001	38
India	5	14	35.7	0.005	39
	15	105	14.3	<0.001	40
	2	39	5.1	<0.001	12
	7	143	4.9	<0.001	10
	1	32	3.1	<0.001	41
	2	77	2.6	<0.001	42
	0	28	0	<0.001	43

^# ^composed of patients with dominant SCA and sporadic ataxia

**Table 3:** Frequencies of MJD in SCA patients from various countries

Countries	Number of MJD	Number of SCA	Frequency (%)	Reference
Portugal	32	38	84.2	16
Brazil	96	114	84.2	17
**Present study**	**100**	**138**	**72.5**	
Portugal/Brazil	67	106	63.2	44
Germany	32	77	41.6	45
France	25	87	28.7	46
Holland	64	227	28.2	47
America*	31	149	20.8	48
Spain	11	72	15.3	49
Australia	11	88	12.5	13
Mexico	13	108	12.0	50
South Africa	2	54	3.7	51
Italy	2	183	1.1	52
UK	0	22	0	53
Serbia	0	38	0	54
Finland	0	49	0	55
Norway	0	19	0	56
Czech	0	118^#^	0	11
Combined	486	1687	28.8	

*The patient subjects mixed with African American, Caucasian American, Cambodian, Japanese, Pakistani, Lebanese and Peruvian; # composed of patients with dominant SCA and sporadic ataxia

**Figure 5:**
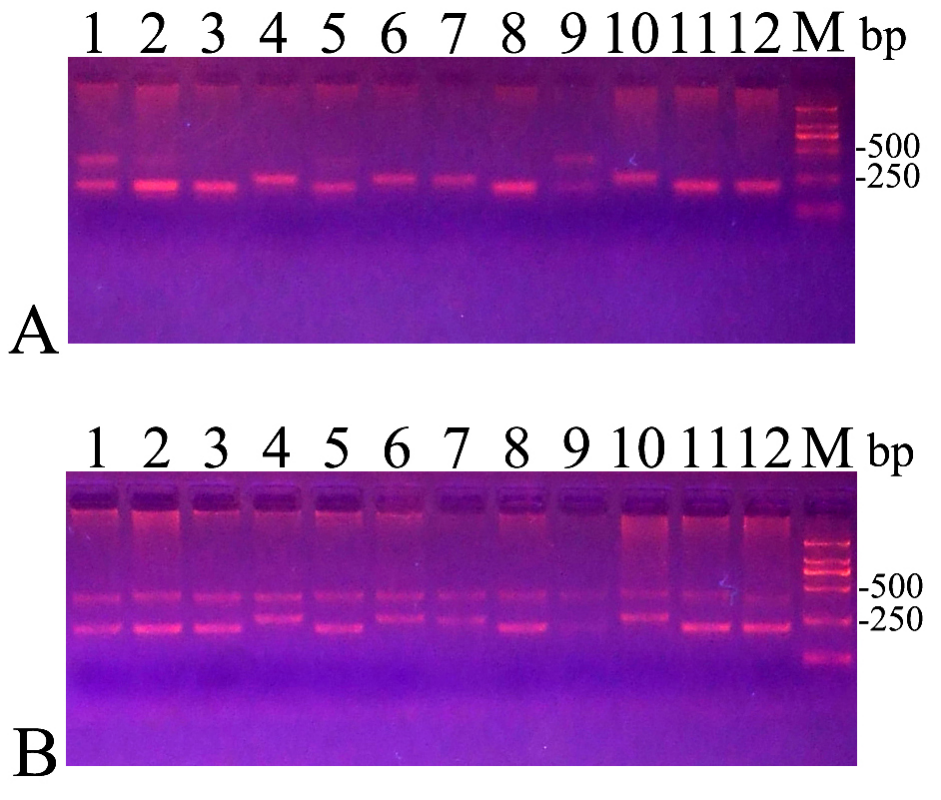
**The agarose gel electrophoresis analysis of alleles of MJD patients**. Lanes 1-12 were all MJD patients and were the same patients in A and B. A: The alleles were amplified with Taq polymerase. B: The alleles were amplified with LA Taq polymerase. M: DL2000 marker.

The agarose gel electrophoresis analysis of alleles of 12 MJD patients is shown in figure 5. All normal alleles were able to be amplified in PCR with La Taq or Taq polymerase. However, reaction efficiency was different for expanded alleles. All 12 and only 2 expaneded alleles were able to be amplified in PCR with La Taq polymerase (figure. 5B) and Taq polymerase (figure. 5A), respectively, for the same 12 MJD patients.

## Discussion

In the present study, we have analyzed the characteristics of CAG repeats of *ATXN3 *in 300 chromosomes of healthy Chinese individuals and found that the CAG repeat number ranged from 12 to 42. The most common allele contained 14 CAG repeats, which was also the most common allele found in the studies of Limprasert et al [21] and Takano et al [9]. The frequency of large ANs (>27 repeats) was 0.28, which is higher than the frequency in previously studied populations [9-11,21].

The frequency of MJD in the 138 SCA families involved in our study was 72.5%, which is, as far as we know, the highest rate in Asia based on studies of the Chinese mainland individuals [22-25], Chinese Taiwanese [26,27], Singaporean [28], Japanese [9,29-35], Thais[36] Korean [37,38] and Indian [10,12,39-43]. Moreover, it is one of the highest worldwide, with only Portuguese and Brazilian populations exhibiting higher proportions [11,13,16,17,44-56]. We presume that there are two possibilities why the relative prevalence of MJD is so high in the present study.

The first reason is that the frequency of large ANs in the present study is very high. Some studies [8-15] suggest that the frequency of normal ANs with a relatively large number of CAG repeats is related to the prevalence of the dominant SCA (including MJD), since the large ANs share the same haplotypes as those of expanded alleles and therefore might act as a reservoir for expanded alleles. However, some other studies reported the prevalence of MJD is not an indirect reflection of the frequency of large normal alleles in Portuguese [57,58]. Here, the frequencies of large ANs are significantly higher than in related data reported by Limprasert et al [21] in >27, >28, and >29 repeats, Takano et al [9] in >28, >29, and >30 repeats, Chattopadhyay et al [10] and Bauer et al [11] in >27, >28, >29, >30, and >31 repeats. In fact, the relative frequency of MJD in the present study was the highest of any published data other than those from Portugal and Brazil, and was significantly higher than in the study of Takano et al [9], Chattopadhyay et al [10] and Bauer et al [11]. As such, the high frequency of large ANs associated with such a high relative frequency of MJD suggests that in this population large ANs may constitute a reservoir from which the expanded alleles may be emerging.

Our use of LA Taq polymerase to amplify alleles of SCA patients is another possible reason for the result. Arezi et al [6] reported that the efficiency of amplification using Taq polymerase was decreased with an increase in amplicon length and GC content, and the reason for the decrease may be related to the enzyme's lack of proofreading activity. Thus, it is difficult to amplify expanded alleles (GC-rich) and sometimes it is impossible to amplify expanded alleles when expanded alleles have a large quantity of CAG repeats [7]. We amplified expanded alleles of the same 12 MJD patients with Taq polymerase and LA Taq polymerase respectively and found most expanded alleles were non-amplified in PCR with Taq polymerase whereas all expanded alleles were amplified in PCR with LA Taq polymerase (figure. 5). For all the normal alleles that were amplified in PCR with Taq polymerase, it was possible that there were false negative results in molecular testing of MJD. The relative frequency of MJD in the present study is significantly higher than in the previous studies that also investigated Chinese SCA patients [22-28]. Notably, in these previous studies, the expanded alleles were amplified using Taq polymerase rather than LA Taq polymerase. Therefore, it may be better to use DNA polymerases that are better at amplifying GC-rich sequences, such as LA Taq, when amplifying the alleles of SCA patients. Additionally, 6 out of the 38 probands with no expanded allele were homozygous for normal alleles. It is possible that these results are false negative results because of either extremely large repeat size or the presence of polymorphisms in the primer-annealing regions [7]. Therefore we need to apply to other techniques such as Southern blot to exclude this possibility of false negative results in further study. However, no matter whatever the results of this further study show, the high relative frequency of MJD in the present study is not affected.

In addition, Martins et al [59] concluded that MJD might first occur in Asia and extend to Europe later, reaching its high prevalence in Portuguese due to founder effect. The high prevalence of MJD (19.2 per 100,000 inhabitants) restricted to the Gosei area of Toyama in Japan [60] may support this conclusion [59]. Therefore we suppose that possible Asian origin of MJD and founder effect may also contribute to the high relative frequency of MJD in the present study.

## Conclusion

We have reported the characteristics of CAG repeats in ATXN3 in a normal Chinese population and in patients with SCA. We found the highest relative frequency of MJD observed in Asian SCA patients. The results support the hypothesis that in this population large ANs may constitute a reservoir from which the expanded alleles may be emerging. Furthermore, LA Taq polymerase was proven to be more efficient than Taq polymerase in the amplification of the expanded alleles, facilitating and improving the molecular diagnosis.

## Competing interests

The authors declare that they have no competing interests.

## Authors' contributions

SRG carried out the molecular genetic studies, participated in the sequence alignment and drafted the manuscript. JJW carried out the molecular genetic studies and participated in the sequence alignment. NW, SXM and CZL participated in analysis and interpretation of data. SSS and STW participated in the sequence alignment. ZYW designed and supervised the study, and critically revised the manuscript for important intellectual content. All authors read and approved the final manuscript.

## Pre-publication history

The pre-publication history for this paper can be accessed here:

http://www.biomedcentral.com/1471-2350/11/47/prepub
